# Clinical Outcomes of Ileostomy Closure during versus after Adjuvant Chemotherapy in Patients with Rectal Cancer

**DOI:** 10.1155/2024/2410643

**Published:** 2024-03-20

**Authors:** Fan He, Fuyu Yang, Chenglin Tang, Defei Chen, Dongqin Zhao, Junjie Xiong, Yu Zou, Guoquan Huang, Kun Qian

**Affiliations:** ^1^Department of Gastrointestinal Surgery, The First Affiliated Hospital of Chongqing Medical University, Chongqing 400016, China; ^2^Hubei Provincial Key Lab of Selenium Resources and Bioapplications, No. 158 Wuyang Avenue, Enshi 445000, Hubei, China

## Abstract

**Background:**

Protective ileostomy can effectively prevent severe anastomotic leakage after rectal cancer surgery; however, the optimal timing for ileostomy closure during adjuvant chemotherapy remains unclear. This study aimed to explore the safety and long-term outcomes of early ileostomy closure during adjuvant chemotherapy.

**Method:**

Patients who underwent laparoscopic rectal cancer surgery combined with protective ileostomy and adjuvant chemotherapy between April 2017 and April 2021 were retrospectively evaluated. Patients were divided into an early closure group during chemotherapy (group A) and a late closure group after chemotherapy (group B).

**Results:**

A total of 215 patients were included in this study, with 115 in group A and 100 in group B. There were no significant differences in demographic and clinical characteristics between the two groups. In group A, durations of stoma status (*p* < 0.001) and low anterior resection syndrome (LARS) (*p* < 0.001) were shorter, and rectal stenosis (*p*=0.036) and stoma-related complications (*p*=0.007), especially stoma stenosis (*p*=0.041), were less common. However, compliance with chemotherapy was worse (*p*=0.009). There were no significant differences in operative time, postoperative hospital stay, postoperative complications, incidence and severity of LARS, disease-free survival, or overall survival between groups.

**Conclusion:**

Early ileostomy closure can effectively reduce the duration of stoma status, duration of LARS, rectal stenosis, and stoma-related complications while not affecting surgical complications and oncological outcomes. Ileostomy closure should not be delayed because of adjuvant chemotherapy. However, follow-up should be strengthened to increase compliance and integrity with chemotherapy.

## 1. Introduction

Radical resection of rectal cancer is the most effective treatment for rectal cancer. To achieve radical resection of the tumor, total mesorectal excision (TME) is key [1], including complete resection of the mesorectum around the rectum and protection of the pelvic autonomic nerve. According to the TME principle, laparoscopic low anterior resection (LAR) for rectal cancer is the mainstream surgical method [2, 3]. With the development of laparoscopy and robot-assisted surgery, it is possible to perform rectal cancer with low or ultralow sphincter-saving surgery [4]. However, owing to the special anatomical position of the rectum, a series of anastomotic-related complications occurred. Anastomotic leakage (AL) is one of the most fearful complications of LAR. The incidence of AL is approximately 1–30% [5, 6], of which 10–35% require reoperation, with a mortality rate of 6–22% [7]. Moreover, some studies [6, 8] have shown that AL after rectal cancer surgery increases local recurrence and long-term mortality.

Protective ileostomy is the most effective surgical method for reducing the occurrence and severity of AL, as confirmed by previous studies [9, 10]. However, this operation has several drawbacks, including several stoma-related complications, such as high-volume output, skin irritation, prolapse, and renal dysfunction. Stoma-related complications may require unplanned hospitalization, resulting in increased quality costs [11]. Moreover, long-term stoma status affects patients' quality of life (QoL) and psychological disorders [12].

Studies [13] have shown that the ideal time interval between primary surgery and closed protective ileostomy is 12 weeks, as this interval allows the patient to fully recover from rectal cancer surgery. However, owing to the use of adjuvant chemotherapy, there is currently no consensus on the optimal timing for protective ileostomy closure. Some studies [14–16] have shown that returning the stoma before adjuvant chemotherapy has the same oncologic outcome as during and even after adjuvant chemotherapy and is beneficial for intestinal function recovery, reducing postoperative nausea, vomiting, and symptoms of low anterior resection syndrome (LARS). However, some researchers [17–19] have contrasting views. They believed that ileostomy closure increased the risk of surgery before or during chemotherapy. Surgical complications affect the integrity of chemotherapy, leading to a delay or cessation of chemotherapy and affecting the efficacy of chemotherapy and long-term prognosis.

Given the controversy over the optimal timing of protective ileostomy closure during chemotherapy, this study aimed to evaluate the safety and long-term prognosis of early ileostomy closure during adjuvant chemotherapy compared to closure after adjuvant chemotherapy.

## 2. Methods

### 2.1. Patients

All patients were diagnosed with rectum adenocarcinoma and underwent laparoscopic rectal cancer surgery combined with protective ileostomy and adjuvant chemotherapy from April 2017 to April 2021. Ileostomy was closed within 1 year of rectal cancer surgery. All patients received adjuvant chemotherapy after rectal cancer surgery, which mainly comprised capecitabine monotherapy and oxaliplatin combined with capecitabine chemotherapy. Among them, oral capecitabine monotherapy mainly included individual patients <30 years old with pathological TNM stage I combined with high-risk factors, part of patients with stage IIa, and patients >80 years old with stage IIb and high-risk factors. This retrospective study did not require informed consent from patients and was approved by the Ethics Review Committee of the hospital.

The exclusion criteria were as follows: 1. stage IV rectal cancer; 2. severe AL after primary surgery; 3. failure of stoma closure; 4. did not receive adjuvant chemotherapy.

In our center, there are two common treatment options for ileostomy closure: restoration of the stoma after 2–3 cycles of adjuvant chemotherapy and restoration of the stoma after completion of adjuvant chemotherapy. Patients were divided into two groups according to the relationship between ileostomy closure and adjuvant chemotherapy. The early closure group (group A) underwent ileostomy closure during chemotherapy, whereas the late closure group (group B) underwent ileostomy closure after a full cycle of chemotherapy. All patients receiving neoadjuvant chemotherapy received 2 cycles of preoperative chemotherapy and were scheduled to receive another 4–6 cycles of adjuvant chemotherapy with the same regimen after surgery. For patients who did not receive neoadjuvant therapy, 6 cycles of chemotherapy were routinely completed after surgery.

Patients who are to close the ileostomy will be evaluated for patency of the anastomosis and safety of ileostomy closure by colonoscopy, abdominal enhanced computed tomography (CT), barium enema, and other imaging examinations preoperatively. All ileostomy closure surgeries were performed via laparotomy, and lateral anastomosis with a stapler was used to reconstruct the digestive tract. All surgeons were associate chief physicians with sufficient surgical experience at the center.

### 2.2. Definition

Ileostomy closure failure was defined as failure to restore the ileostomy after more than 12 months following primary surgery.

Anastomotic stenosis (AS) was defined as the inability of a probe with a diameter of 12 mm to pass through the anastomosis during preostomy reduction colonoscopy or the inability of the finger to pass through the anastomosis in a digital rectal examination [20].

LARS was defined as rapid bowel movement, frequent bowel movements, difficulty in emptying, and fecal incontinence after rectal surgery [21].

Stoma stenosis was defined as a narrowing of the stoma opening, resulting in the inability of the stoma to discharge properly [22].

### 2.3. Outcomes

The parameters for comparison included demographic characteristics, rectal cancer characteristics, interval between primary surgery and ileostomy closure, number of overall chemotherapy cycles, time to first flatus, time to full oral nutrition, postoperative hospital stay, postoperative complications, and stoma-related complications of the two groups. The incidence, severity, and duration of LARS after ileostomy closure as well as the disease-free survival (PFS) and overall survival (OS) of patients were assessed. Patient data during hospitalization were extracted from the electronic medical record system, and stoma-related complications were extracted from cases filled in by the stoma nurse. The number of chemotherapy cycles was determined by referring to hospital records or telephone follow-ups. PFS and OS were obtained by referring to previous inpatient and outpatient medical records for auxiliary examinations and telephone follow-ups. The follow-up period was from the time of surgery to June 2023. LARS status was obtained via telephone follow-up and LARS questionnaire score. The LARS rating questionnaire mainly includes the following questions: “incontinence for flatus,” “incontinence for liquid stool,” “frequency of bowel movements,” “clustering of stools,” and “urgency.” The score range was from 0 to 42, with limits of 0–20 (no LARS), 21–29 (minor LARS), and 30–42 (major LARS) [21].

### 2.4. Statistical Analysis

SPSS version 27.0 (IBM Corp.; Armonk, NY, USA) was used for statistical analysis. A two-sided *p* value <0.05 was considered statistically significant. GraphPad Prism software (version 9.0) was used to construct the survival curves. Continuous variables with normal distribution were represented by mean and standard deviation using Student's *t*-test, while continuous variables with nonnormal distribution were represented by median and quartiles using the Mann–Whitney *U* test. Categorical variables were represented by frequency (%), and the Chi-square test and Fisher's exact test were conducted. Intergroup survival curves for DFS and OS were calculated using Kaplan–Meier analysis and compared using the logarithmic rank test. Subgroup analysis was used to exclude possible confounding factors to verify the reliability and stability of the study results.

## 3. Results

**Figure 1:**
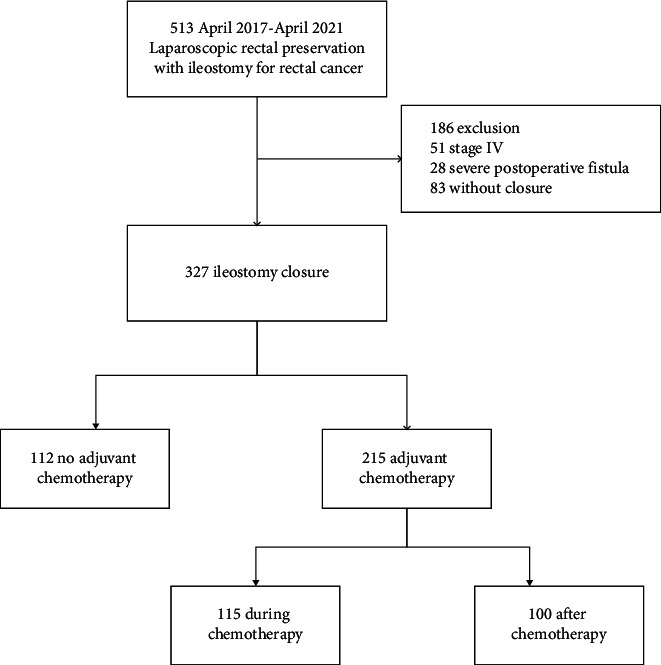
Flowchart of the patients included in this study.

We retrospectively evaluated 513 patients who underwent laparoscopic radical resection for rectal cancer combined with protective ileostomy between April 2017 and April 2021. Of these patients, 51 had distant metastasis, 28 had severe AL after rectal cancer surgery, 83 failed to restore the ileostomy, and 112 did not receive adjuvant chemotherapy. After excluding these patients, 215 patients (115 in group A and 100 in group B) were finally analyzed (Figure 1). No significant differences were discovered in the demographic and clinical characteristics between the two groups (Table 1).

There were no significant differences in sex, age, body mass index (BMI), smoking, alcohol consumption, comorbidities, American Society of Anesthesiologists (ASA) score, tumor location, tumor node metastasis (TNM) stage, neoadjuvant chemoradiotherapy (NCRT), or ileostomy location between the two groups.

The duration of stoma status in the early closure group was significantly less than that in the late closure group (96.03 ± 18.15 vs. 191.64 ± 57.92, *p* < 0.001). As for the safety of stoma closure surgery, there were no significant differences between the two groups for operation time, intraoperative blood loss, first postoperative exhaust time, postoperative hospital stay, or postoperative complications (*p* > 0.05). There were also no statistical differences in creatinine increase and BMI decrease between groups (*p* > 0.05).

The incidence of rectal AS was observably lower in the early closure group (12.2% vs. 23%, *p*=0.036). The main methods of evaluating the anastomosis were colonoscopy and CT, and no significant differences were discovered in the evaluation methods between groups (*p* > 0.05). No significant difference in disuse colitis was observed between groups (*p*=0.971). During the entire course of adjuvant chemotherapy, there was no significant difference in chemotherapy regimen between the two groups; however, chemotherapy compliance was better in the late closure group (5.19 ± 1.53 vs. 5.72 ± 1.41, *p*=0.009) (Table 2).

Stoma-related complications were markedly less common in the early closure group (13.9% vs. 29%), demonstrating a statistically significant difference (*p*=0.007), especially in stoma stenosis (2.6% vs. 10%, *p*=0.041). There was no significant difference among patients with skin irritation, parastomal hernia, or ileus caused by stoma (*p* > 0.05). Among all follow-up patients, 3 (2.6%) and 2 (2%) patients in the early and late closure groups, respectively, underwent reostomy because of intestinal fistula. Moreover, 1 (0.9%) and 2 (2.0%) patients in the early and late closure groups, respectively, underwent reostomy owing to severe LARS (Table 3).

**Figure 2:**
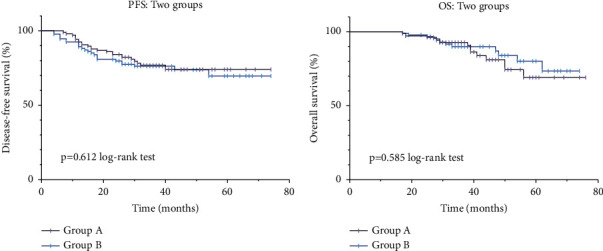
(a) Survival curve of overall PFS. (b) Survival curve of overall OS.

**Figure 3:**
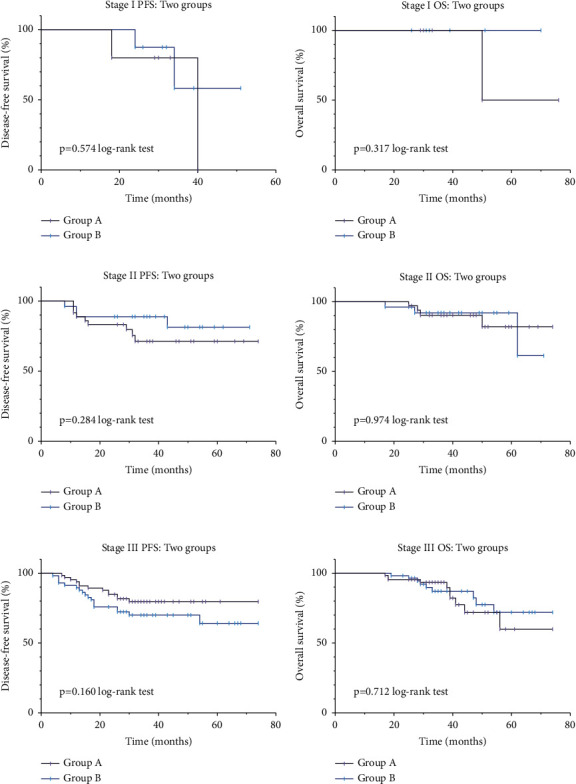
(a) Survival curve of stage I PFS. (b) Survival curve of stage I OS. (c) Survival curve of stage II PFS. (d) Survival curve of stage II OS. (e) Survival curve of stage III PFS. (f) Survival curve of stage III OS.

In terms of long-term prognosis, 201 of the 215 patients completed the follow-up period and were included in the survival analysis. No significant differences were discovered in the follow-up time between groups (*p*=0.393) and no significant difference in PFS and OS (*p*=0.612 and *p*=0.585, respectively) (Figure 2). Survival analysis was also conducted for tumors with different clinical stages; the results showed that in patients with rectal cancer with clinical stages I, II, and III, no significant statistical differences were found in PFS and OS at different ileostomy closure timings (*p*=0.574 and *p*=0.317, *p*=0.284 and 0.974, and *p*=0.160 and 0.712, respectively) (Figure 3).

Of the 201 patients who completed the follow-up, 174 completed the LARS questionnaire via telephone inquiry. There was no significant difference in the incidence and severity of LARS between groups (*p*=0.690); however, the duration of LARS was shorter in the early closure group (*p* < 0.001). No significant difference was found in “incontinence for flatus,” “incontinence for liquid stool,” “frequency of bowel movements,” and “clustering of stools” between groups although the urgency symptoms were significantly more severe in the late closure group (*p*=0.032) (Table 4).

### 3.1. Subgroup Analysis

We performed a subgroup analysis for patients who did not receive NCRT to verify the reliability of the findings. After excluding patients receiving NCRT, there were no significant statistical differences between the two groups in sex, age, ASA score, tumor location, and other baseline characteristics (Supplementary Table 1). Consistent with the results before subgroup analysis, we found that in terms of the safety of closure surgery, there were no significant differences in the amount of intraoperative blood loss, postoperative feeding time, postoperative hospital stay, and postoperative complications among patients with different ileostomy closure timings. The incidence of rectal AS was remarkably lower in the early closure group during adjuvant chemotherapy (13.3% vs. 26.8%, *p*=0.045). However, we did not find a significant difference in chemotherapy compliance between patients who underwent ileostomy closure during and after adjuvant chemotherapy (*p*=0.17) (Supplementary Table 2). In addition, we found significantly fewer stoma-related complications in the early closure group during adjuvant chemotherapy (12.0% vs. 26.8%, *p*=0.026), especially in stoma stenosis (2.4% vs. 12.5%, *p*=0.03) (Supplementary Table 3). After excluding patients who received NCRT, we still found no statistical difference in the long-term prognosis of patients who underwent ileostomy closure at different timings (Supplementary Figure 1). In the subgroup analysis, the postoperative LARS duration was shorter (*p* < 0.001) and the postoperative urgency symptoms were less frequent (*p*=0.044) (Supplementary Table 4).

## 4. Discussion

In this study, we evaluated the safety and long-term prognosis of the early closure of a protective ileostomy during adjuvant chemotherapy. Chemotherapy compliance and severity and duration of LARS after ileostomy closure were also compared. Our results show that temporary ileostomy early closure during adjuvant chemotherapy is safe and feasible and has no significant effect on long-term prognosis. It can achieve similar clinical treatment levels. Moreover, in patients who underwent early closure of the ileostomy during adjuvant chemotherapy, the duration of stoma status and LARS was shorter, and the rectal AS and stoma-related complications were lower. However, early ileostomy closure during chemotherapy worsens compliance with chemotherapy. In future clinical work, the follow-up of such patients should be strengthened to increase the compliance and integrity of adjuvant chemotherapy.

Protective ileostomy has caused some negative impacts on patients, including stoma-related complications, psychosocial influences, and obstacles pertaining to sexual life [12, 23]. For most patients with rectal cancer, postoperative adjuvant chemotherapy leads to better oncology outcomes [24]. Thalheimer et al. [25] suggest that closing the stoma before adjuvant chemotherapy leads to delayed chemotherapy and higher complication rates. Possible reasons for this include the fact that the body is generally damaged during chemotherapy, as well as reduced tolerance and healing ability. However, Choi et al. [26] believe that ileostomy closure during chemotherapy is clinically safe and that chemotherapy interruption due to ileostomy reduction does not alter oncological outcomes. Cheng et al. [27] came to the same conclusion, suggesting no increase in surgical complications among patients who received chemotherapy after rectal cancer surgery. In this study, we also reached the same point of view, and no significant difference was observed between the two groups of patients in terms of postoperative complications, which proves the safety and feasibility of undergoing reductive surgery during chemotherapy.

We found no significant differences in operation time, first exhaust defecation time, intraoperative blood loss, or postoperative hospital stay between the two groups. These results were similar to those of a meta-analysis by Hajibandeh et al. [28]. Adjuvant chemotherapy has no obvious effect on ileostomy closure surgery, and ileostomy is safe to restore during chemotherapy. Moreover, the results of this study showed no significant difference in hospitalization costs between the two groups studied. However, early restoration of the stoma could reduce the cost of stoma care, reducing the economic pressure and burden on patients [29].

We revealed that the rectal AS was higher in the late closure group (*p*=0.036), which is consistent with the results of the study of Zhang et al. [30]. The study of Babayev et al. [31] also showed that prolonged ileostomy increases the incidence of AS. A possible reason is that the expansion of the intestinal tract stimulated by the presence of feces helps to prevent AS; protective ileostomy causes the diversion of feces, and anastomosis loses this preventive stimulation effect, increasing the risk of AS [32, 33]. The presence of a protective stoma reduces the movement of the distal intestine, which is a mechanism that increases AS risk [34]. Therefore, early closure of the ileostomy can help to reduce rectal AS, and the prolongation of the duration of stoma status and increase in the cost of treatment due to AS should be avoided.

In addition, in the early closure group, patients had worse chemotherapy compliance, which may be because before the patients underwent ileostomy closure, feces were discharged through the stoma without anal discomfort. After stoma restoration, most patients will experience LARS [21], which increases patient pain, deteriorates physical condition, and decreases the ability to tolerate chemotherapy, leading to a decline in chemotherapy compliance for some patients. Therefore, for patients undergoing early ileostomy closure during chemotherapy, publicity and education should be strengthened before restoration surgery and discharge after surgery, patients should be informed of possible LARS, and follow-up should be strengthened to reduce the effect of adjuvant chemotherapy deficiency on long-term oncological outcomes.

Studies [14] have shown that a few stoma-related complications in long-term stoma status affect patient QoL, and early ileostomy closure can reduce stoma-related complications [35]. This finding is consistent with the results of the present study. In the early closure group, the duration of stoma status was shorter, and stoma-related complications, especially stoma stenosis, were significantly less frequent. Stoma stenosis is a long-term complication, the main mechanism of which is ostomy ischemia [36]. Patients with stoma stenosis are prone to acute ileus, which can seriously endanger life. Regular stoma dilation is recommended in patients with long-term ileostomy persistence. However, early return to ileostomy can reduce the occurrence of root stenosis.

No significant differences were found in PFS and OS between groups, indicating that although chemotherapy compliance is poor in early ileostomy closure during adjuvant chemotherapy, it still does not affect long-term prognosis, and a little delay or suspension of chemotherapy due to ileostomy closure does not affect oncological outcomes. However, some experts give up early restoration of the protective ileostomy owing to fear of postoperative complications, affecting the process of chemotherapy, and choose to perform stoma closure after chemotherapy [25, 37]. In this study, only 2 patients (1.7%) developed AL postoperatively and were cured and discharged from the hospital by conservative treatment, including antiinfection and surgical drainage, delaying the longer chemotherapy process. Most patients can complete chemotherapy on schedule. Therefore, there is a lower chance of an excessive delay in chemotherapy due to complications of stoma closure surgery [38]. However, the median follow-up time of patients included in this study was short, and the long-term prognostic value of this study was limited. In the future, we will continue to follow-up patients for PFS and OS to confirm the reliability of the results of this study.

LARS comprises a series of symptoms that occur after rectal surgery. The symptoms were the most intense in the early postoperative period, lasting approximately 3 months, and subsequently gradually relieved and entered a stable phase after 1-2 years. Some severe cases of LARS persisted for life. Several studies have explored the incidence, influencing factors, and treatment of rectal LARS following rectal surgery [39–41]. However, few studies have investigated the association between stoma duration and incidence, severity, and duration of LARS. Studies [14] have shown that a prolonged ileostomy closure time seems to negatively impact intestinal function. The long-term persistence of a stoma may lead to distal digestive tract atrophy, shunt colitis, and changes in intestinal flora.

The results of this study found no significant differences in the incidence and severity of disused colitis and LARS between the two groups. However, the duration of LARS was significantly shorter in the early closure group. Postoperative QoL significantly improved, especially in terms of urgency. The urgency of postoperative stools was less frequent, which reduced the distress caused by urgent stools when patients went out. We also found that among the patients who were followed up, only a few were treated with LARS. After rectal surgery, surgeons should strengthen follow-up and provide suggestions to patients to improve their defecation symptoms and QoL.

Studies [33, 42, 43] have shown that NCRT may increase anastomosis-related complications after rectal cancer surgery, especially AL and AS. Moreover, it may also change the microenvironment around the rectum, increase inflammatory response, and increase postoperative complications. Therefore, we excluded patients who received NCRT and performed subgroup analyses to improve the reliability and stability of the findings. The results showed that the outcomes did not change significantly after excluding patients receiving NCRT. However, for patients who only received adjuvant chemotherapy after surgery, there was no statistically significant difference in chemotherapy compliance among patients with different ileostomy closure timings. On the one hand, it may be due to insufficient sample size in subgroup analysis; on the other hand, part of the patients with NCRT had good treatment effect, leading to the compliance of postoperative adjuvant chemotherapy decreased after the tumor stage descending. However, further randomized controlled trials (RCTs) with larger sample sizes are needed in the future to verify the effect of the timing of ileostomy closure on patients' compliance with adjuvant chemotherapy.

This study performed a preliminary analysis of the number of cycles of adjuvant chemotherapy and the completeness of chemotherapy in patients returning during and after adjuvant chemotherapy. Second, we further analyzed the influence of different stoma status durations on LARS and confirmed the superiority of early ileostomy closure, which has guiding significance for clinical work. However, there are several limitations to this study. First, it was a retrospective cohort study. Second, all patients included in this study received adjuvant chemotherapy (oral capecitabine monotherapy and oxaliplatin combined with capecitabine), excluding patients with distant metastasis. Therefore, the use of targeted therapy in patients with distant metastases is of limited significance. Moreover, there have been a few cases of early ileostomy closure before adjuvant chemotherapy at our center. We failed to explore the surgical safety and long-term prognosis of ileostomy closure before adjuvant chemotherapy. In the future, well-designed and large-sample RCTs are needed to further verify the safety, chemotherapy integrity, long-term prognosis, and LARS in patients undergoing protective ileostomy with early closure during or before adjuvant chemotherapy.

## 5. Conclusion

This study indicates that early ileostomy closure during adjuvant chemotherapy is safe and feasible, reducing stoma-related complications and rectal AS and shortening the duration of stoma status and LARS. Although chemotherapy compliance was worse, it did not change the long-term oncological outcomes. Ileostomy closure should not be delayed because of the need for adjuvant chemotherapy. However, follow-up should be strengthened to enhance chemotherapy compliance and avoid the postponement of adjuvant chemotherapy. Simultaneously, patients should be guided to cope well with LARS and reduce the impact of LARS on QoL through drug and physical therapy by strengthening follow-up.

## Figures and Tables

**Table 1:** Comparison of demographic characteristics between two groups.

	Group A	Group B	*p* value
	(*n* = 115) (%)	(*n* = 100) (%)	
Sex, male	73 (63.5%)	61 (61.0%)	0.708
Age (year)	61.43 ± 10.66	59.77 ± 10.74	0.259
BMI (kg/m^2^)	22.87 ± 3.185	23.33 ± 2.91	0.274
Smoking	46 (40.0%)	48 (48.0%)	0.238
Alcohol consumption	44 (34.8%)	44 (44.0%)	0.393
Comorbidity			
Hypertension	26 (22.6%)	23 (23.0%)	0.946
Diabetes	16 (13.9%)	8 (8.0%)	0.170
Coronary disease	14 (12.2%)	11 (11.0%)	0.789
Pneumonia	6 (5.2%)	9 (9.0%)	0.278
Hepatitis	3 (2.6%)	8 (8.0%)	0.118
ASA score			0.668
I	37 (32.2%)	38 (38.0%)	
II	51 (44.3%)	41 (41.0%)	
III	27 (23.5%)	21 (21.0%)	
cTNM stage			0.475
I	5 (4.3%)	8 (8.0%)	
II	37 (32.2%)	28 (28.0%)	
III	73 (63.5%)	64 (64.0%)	
ypTNM stage			0.917
0	1 (0.9%)	2 (2.0%)	
I	7 (6.1%)	10 (10.0%)	
II	11 (9.6%)	12 (12.0%)	
III	13 (11.3%)	20 (20.0%)	
Neoadjuvant radiotherapy	22 (19.1%)	28 (28.0%)	0.125
Neoadjuvant chemotherapy	32 (27.9%)	36 (36.0%)	0.199
Tumor location (cm)	5.97 ± 2.12	5.52 ± 2.12	0.127
Albumin(g/L)	37.99 ± 4.03	37.79 ± 3.83	0.583
Serum creatinine(*μ*mol/L)	70.97 ± 17.44	70.59 ± 11.14	0.854
Stoma location, right	46 (40.0%)	50 (50.0%)	0.141
Distance between stoma and ileocecal part (cm)	35.33 ± 9.36	33.07 ± 11.38	0.245

BMI, body mass index; ASA, American Society of Anesthesiologists; cTNM, clinical tumor node metastasis; ypTNM, neoadjuvant pathological tumor node metastasis.

**Table 2:** Data related to ileostomy closure.

	Group A	Group B	*p* value
	(*n* = 115), (%)	(*n* = 100), (%)	
Interval to ileostomy closure (day; IQR)	98 (83–110)	179 (152–211)	<0.001^*∗*^
Operation time (min)	87.41 ± 34.29	91.21 ± 37.99	0.442
Blooding loss (mL)	23.77 ± 16.72	25.15 ± 17.89	0.558
Time to first flatus (day)	3.28 ± 1.08	3.22 ± 0.92	0.673
Time to fully oral nutrition (day)	3.30 ± 1.08	3.32 ± 1.13	0.872
Hospital stay (day)	5.17 ± 1.87	5.31 ± 1.68	0.558
Postoperative complications	18/115 (15.7%)	22/100 (22.0%)	0.102
Clavien–Dindo classification			
Grade I	8 (7.0%)	8 (8.0%)	
Grade II	2 (1.7%)	10 (10.0%)	
Grade IIIa	2 (1.7%)	1 (1.0%)	
Grade IIIb	6 (5.2%)	3 (3.0%)	
Chemotherapy regimens			0.686
Monotherapy	22	17	
Combined chemotherapy	93	83	
Postoperative infection	2 (1.7%)	4 (4.0%)	0.315
Cost (yuan)	39613.28 ± 11004.39	37142.76 ± 12427.55	0.124
Increased creatinine (*μ*mol/L)	8.43 ± 31.26	4.95 ± 14.73	0.309
Decreased BMI (kg/m^2^)	0.91 ± 1.71	0.67 ± 1.46	0.275
Anastomotic evaluation method			
Endoscope	99 (86.1%)	91 (91.0%)	0.262
Endoscope + CT	74 (64.3%)	69 (69.0%)	0.471
Rectal anastomosis stenosis	14 (12.2%)	23 (23.0%)	0.036^*∗*^
Disused colitis	29 (25.2%)	25 (25%)	0.971
Chemotherapy cycles (times)	5.19 ± 1.53	5.72 ± 1.41	0.009^*∗*^
Follow-up time (IQR)	36 (28–47)	36 (29–51)	0.393
Postoperative regular follow-up	78 (67.8%)	66 (66.0%)	0.726

IQR, interquartile range; BMI, body mass index; CT, computed tomography; ^*∗*^*p* < 0.05.

**Table 3:** Complications related to ileostomy.

	Group A	Group B	*p* value
	(*n* = 115), (%)	(*n* = 100), (%)	
Total complication	16 (13.9%)	29 (29.0%)	0.007^*∗*^
Skin irritation	5 (4.3%)	8 (8.0%)	0.262
Parastomal hernia	5 (4.3%)	6 (6.0%)	0.758
Stoma stenosis	3 (2.6%)	10 (10.0%)	0.041^*∗*^
Ileus due to stoma	3 (2.6%)	5 (5.0%)	0.477
Ileostomy after stoma closure	4 (3.5%)	4 (4.0%)	1.000

^*∗*^ *p* < 0.05.

**Table 4:** Data for LARS.

	Group A	Group B	*p* value
	(*n* = 92), (%)	(*n* = 82), (%)	
LARS			0.690
No	12 (13.0%)	8 (9.8%)	
Minor	38 (41.3%)	32 (40.0%)	
Major	42 (45.7%)	42 (51.2%)	
LARS lasting time (month; IQR)	12 (8–17)	21 (12–31)	<0.001^*∗*^
Seek medical advice because of LARS	25 (27.2%)	24 (29.3%)	0.759
Incontinence for flatus			0.350
Never	15 (16.3%)	16 (19.5%)	
<Once a week	57 (62.0%)	42 (51.2%)	
≥Once a week	20 (21.7%)	24 (29.3%)	
Incontinence for liquid stools			0.251
Never	20 (21.7%)	10 (12.2%)	
<Once a week	54 (58.7%)	54 (65.9%)	
≥Once a week	18 (19.6%)	18 (22.0%)	
Frequency of bowel movements			0.134
>7 times a day	16 (17.4%)	21 (25.6%)	
4–7 times a day	45 (49.0%)	32 (39.0%)	
1–3 times a day	28 (30.4%)	21 (25.6%)	
<Once a day	3 (3.2%)	8 (9.8%)	
Clustering of stools			0.467
Never	4 (4.3%)	1 (1.2%)	
<Once a week	53 (57.6%)	49 (59.8%)	
≥Once a week	35 (38.0%)	32 (39.0%)	
Urgency			0.032^*∗*^
Never	12 (13.0%)	2 (2.4%)	
<Once a week	57 (62.0%)	54 (65.9%)	
≥Once a week	23 (25.0%)	26 (31.7%)	

LARS, low anterior resection syndrome; IQR, interquartile range; ^*∗*^*p* < 0.05.

## Data Availability

All data generated or analyzed during this study are included in this published article.

## Supplementary Materials

Supplementary Materials Supplementary Table 1: comparison of demographic characteristics between two subgroups. BMI: body mass index; ASA: American Society of Anesthesiologists; cTNM: clinical tumor node metastasis. Supplementary Table 2: date related to ileostomy closure of two subgroups. IQR: interquartile range; BMI: body mass index; CT: computed tomography; ^*∗*^*p* < 0.05. Supplementary Table 3: complication related to ileostomy of two subgroups. ^*∗*^*p* < 0.05. Supplementary Table 4: date for LARS of two subgroups. LARS: low anterior resection syndrome; IQR: interquartile range; ^*∗*^*p* < 0.05. Supplementary Figure 1.
