# Supplementary material for: Clinical Outcomes of Ileostomy Closure during versus after Adjuvant Chemotherapy in Patients with Rectal Cancer
Source: Can J Gastroenterol Hepatol. 2024 Mar 20;2024:2410643. doi: 10.1155/2024/2410643 (PMC10977340; doi:10.1155/2024/2410643)

| **Supplementary Table1: Comparison of demographic characteristics between two subgroups.** | | | |
| --- | --- | --- | --- |
|  | **Subgroup A** | **Subgroup B** | **p-value** |
|  | **(n=83) %** | **(n=56) %** |  |
| **Sex, male** | 49(59.0%) | 32(57.1%) | 0.824 |
| **Age (year)** | 61.69±11.70 | 61.21±10.39 | 0.808 |
| **BMI (kg/m^2^)** | 23.07±3.27 | 23.44±3.04 | 0.510 |
| **Smoking** | 31(37.3%) | 26(46.4%) | 0.286 |
| **Alcohol consumption** | 31(37.3%) | 28(50.0%) | 0.139 |
| **Comorbidity** |  |  |  |
| Hypertension | 19(22.9%) | 11(19.6%) | 0.648 |
| Diabetes | 10(12.0%) | 7(12.5%) | 0.936 |
| Coronary disease | 10(12.0%) | 7(12.5%) | 0.936 |
| Pneumonia | 4(4.8%) | 6(10.7%) | 0.187 |
| Hepatitis | 2(2.4%) | 6(10.7%) | 0.061 |
| **ASA score** |  |  | 0.738 |
| I | 30(36.1%) | 17(30.3%) |  |
| II | 35(42.2%) | 27(48.2%) |  |
| III | 18(21.7%) | 12(21.4%) |  |
| **cTNM stage** |  |  | 0.411 |
| I | 4(4.8%) | 6(10.7%) |  |
| II | 33(39.8%) | 20(35.7%) |  |
| III | 46(55.4%) | 30(53.6%) |  |
| **Tumor location (cm)** | 5.67±1.85 | 5.52±2.32 | 0.676 |
| **Albumin(g/L)** | 38.01±4.29 | 37.52±4.09 | 0.499 |
| **Serum creatinine(umol/L)** | 70.30±15.86 | 71.11±11.16 | 0.743 |
| **Stoma location, right** | 33(39.8%) | 30(53.6%) | 0.109 |
| **Distance between stoma and ileocecal part (cm)** | 35.59±9.65 | 32.31±11.33 | 0.174 |

BMI,: body mass index; ASA,: American Society of Anesthesiologists; cTNM,: clinical tumor node metastasis;

| **Suppmentary Table2: Date related to ileostomy closure of two subgroups.** | | | |
| --- | --- | --- | --- |
|  | **Subgroup A** | **Subgroup B** | **p-value** |
|  | **(n=83),%** | **(n=56),%** |  |
| **Interval to ileostomy closure (day; IQR）** | 96(83-110) | 182(150-216) | <0.001* |
| **Operation time(min)** | 82.41±30.16 | 93.45±38.03 | 0.059 |
| **Blooding loss(mL)** | 22.87±15.56 | 25.36±16.97 | 0.374 |
| **Time to first flatus(day)** | 3.33±1.06 | 3.27±0.84 | 0.735 |
| **Time to fully oral nutrition(day)** | 3.30±1.07 | 3.27±1.05 | 0.856 |
| **Hospital stay(day)** | 5.28±1.89 | 5.32±1.78 | 0.890 |
| **Postoperative complications**  **Clavien-Dindo classification** | 12/83(14.5%) | 11/56(19.6%) | 0.344 |
| Garde I | 7(8.4%) | 5(8.9%) |  |
| Garde II | 2(2.4%) | 5(8.9%) |  |
| Garde IIIa | 0(0%) | 0(0%) |  |
| Garde IIIb | 3(3.6%) | 1(1.8%) |  |
| **Chemotherapy regimens** |  |  | 0.839 |
| Monotherapy | 19 | 12 |  |
| Combined chemotherapy | 64 | 44 |  |
| **Postoperative infection** | 1(1.7%) | 2(3.6%) | 0.146 |
| **Cost(yuan)** | 40013.29±10729.94 | 39040.64±14670.17 | 0.652 |
| **Increased creatinine(umol/L)** | 3.86±18.40 | 5.91±17.79 | 0.514 |
| **Decreased BMI(kg/m^2^)** | 0.96±1.64 | 0.64±1.66 | 0.270 |
| **Anastomotic evaluation method** |  |  |  |
| **Endoscope** | 69(83.1%) | 53(94.6%) | 0.063 |
| **Endoscope+CT** | 52(62.7%) | 39(69.6%) | 0.395 |
| **Rectal anastomosis stenosis** | 11(13.3%) | 15(26.8%) | 0.045* |
| **Disused colitis** | 21(25.3%) | 13(23.2%) | 0.779 |
| **Chemotherapy cycles(times)** | 5.02±1.67 | 5.39±1.27 | 0.170 |
| **Follow-up time (IQR)** | 35.5(28-47) | 38.5(27-54) | 0.313 |
| **Postoperative regular follow-up** | 63(75.9%) | 38(67.9%) | 0.615 |

IQR,: interquartile range; BMI,: body mass index; CT,: computed tomography; *: p<0.05

| **Suppmentary Table3: Complication related to ileostomy of two subgroups.** | | | |
| --- | --- | --- | --- |
|  | **Subgroup A** | **Subgroup B** | **p-value** |
|  | **(n=83),%** | **(n=56),%** |  |
| **Total complication** | 10(12.0%) | 15(26.8%) | 0.026* |
| **Skin irritation** | 4(4.8%) | 4(7.1%) | 0.714 |
| **Parastomal hernia** | 3(3.6%) | 3(5.4%) | 0.685 |
| **Stoma stenosis** | 2(2.4%) | 7(12.5%) | 0.030* |
| **Ileus due to stoma** | 3(3.6%) | 2(3.6%) | 1.000 |
| **Ileostomy after stoma closure** | 1(1.2%) | 0(0%) | 1.000 |

*: p<0.05

| **Suppmentary Table4: Date for LARS of two subgroups** | | | |
| --- | --- | --- | --- |
|  | **Subgroup A** | **Subgroup B** | **p-value** |
|  | **(n=68),%** | **(n=47),%** |  |
| **LARS** |  |  | 0.453 |
| No | 9(13.2%) | 3(6.4%) |  |
| Minor | 28(41.2%) | 19(40.4%) |  |
| Major | 31(45.6%) | 25(53.2%) |  |
| **LARS lasting time(month; IQR)** | 12(9-17) | 21(12-31) | <0.001* |
| **Seek medical advice due to LARS** | 18(26.4%) | 13(27.7%) | 0.888 |
| **Incontinence for Flatus** |  |  | 0.819 |
| Never | 10(14.7%) | 9(19.1%) |  |
| <once a week | 44(64.7%) | 29(53.2%) |  |
| ≥once a week | 14(20.6%) | 9(19.1%) |  |
| **Incontinence for liquid stools** |  |  | 0.455 |
| Never | 14(20.6%) | 6(12.8%) |  |
| <once a week | 42(61.8%) | 34(72.3%) |  |
| ≥once a week | 12(17.6%) | 7(14.9%) |  |
| **Frequency of bowel movements** |  |  | 0.176 |
| >7 times a day | 10(14.7%) | 12(25.5%) |  |
| 4-7 times a day | 36(52.9%) | 17(36.2%) |  |
| 1-3 times a day | 19(27.9%) | 13(27.7%) |  |
| <once a day | 3(4.4%) | 5(10.6%) |  |
| **Clustering of stools** |  |  | 0.376 |
| Never | 2(2.9%) | 0(0%) |  |
| <once a week | 39(57.4%) | 31(66.0%) |  |
| ≥once a week | 27(39.7%) | 16(34.0%) |  |
| **Urgency** |  |  | 0.044* |
| Never | 11(16.2%) | 1(2.1%) |  |
| <once a week | 39(57.4%) | 34(72.3%) |  |
| ≥once a week | 18(26.5%) | 12(25.5%) |  |

LARS: low anterior resection syndrome; IQR: interquartile range; *: p<0.05

**Supplementray Figure 1.**


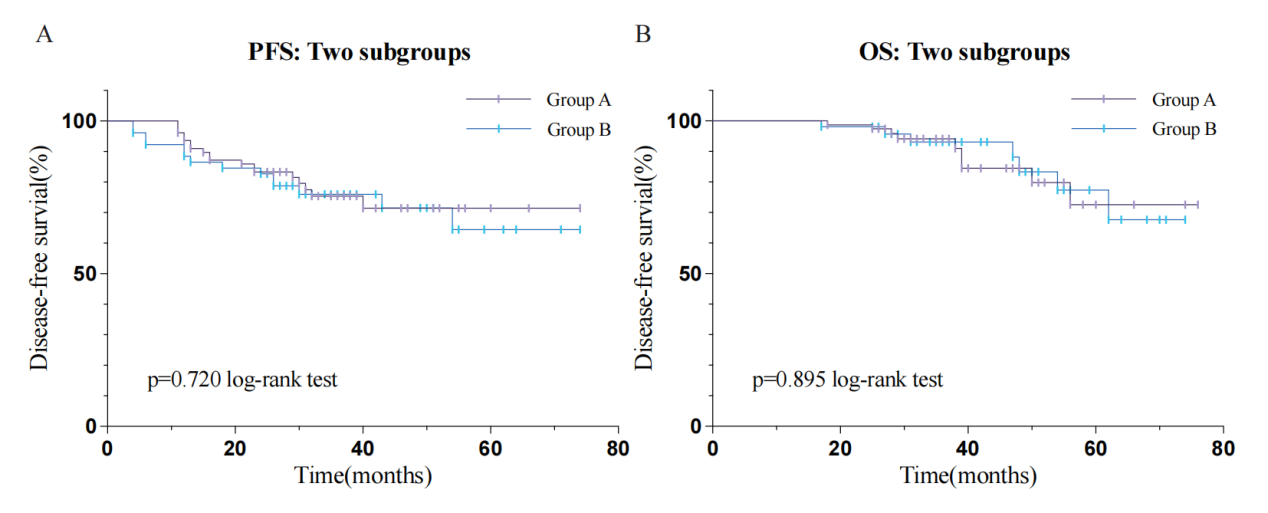

Supplement: Supplementary Materials — Supplementary Table 1: comparison of demographic characteristics between two subgroups. BMI: body mass index; ASA: American Society of Anesthesiologists; cTNM: clinical tumor node metastasis. Supplementary Table 2: date related to ileostomy closure of two subgroups. IQR: interquartile range; BMI: body mass index; CT: computed tomography; ∗p < 0.05. Supplementary Table 3: complication related to ileostomy of two subgroups. ∗p < 0.05. Supplementary Table 4: date for LARS of two subgroups. LARS: low anterior resection syndrome; IQR: interquartile range; ∗p < 0.05. Supplementary Figure 1. [file 2410643.f1.docx]
